# Amplification of phosphoglycerate dehydrogenase diverts glycolytic flux and contributes to oncogenesis

**DOI:** 10.1186/1753-6561-6-S3-O15

**Published:** 2012-06-01

**Authors:** Jason W Locasale, Alexandra Grassian, Rameen Beroukhim, Matthew Meyerson, Gerhard Wagner, John M Asara, Joan S Brugge, Matthew G Vander Heiden, Lewis C Cantley

**Affiliations:** 1 Harvard Medical School, Boston, MA, USA; 2 Department of Medicine, Division of Signal Transduction, Beth Israel Deaconess Medical Center, Boston, MA, USA; 3 Koch Institute for Integrative Cancer Research, Department of Biology, Massachusetts Institute of Technology, Cambridge, MA, USA

## 

Most tumors display enhanced glucose metabolism compared to normal tissues [1-6]. The preferential conversion of glucose to lactate in cancer cells (the Warburg Effect) was one of the first known differences between tumor and normal cells and is believed to contribute to enhanced growth in tumor cells. However, the extent to which specific metabolic fluxes originating from glucose branch from central carbon metabolism and are utilized for anabolic processes is poorly understood. Here, we used an integrated, quantitative metabolomics approach combining NMR experiments with heavy isotope labeling and targeted mass-spectrometry. We carried out direct measurements of metabolic fluxes emanating from glucose metabolism [7].

We found that in some cancer cells, a relatively large amount of glycolytic carbon was diverted into serine and glycine biosynthesis. Serine can be synthesized from a glycolytic intermediate, 3-phosphoglycerate (3PG). 3PG is oxidized by an enzyme phosphoglycerate dehydrogenase (PHGDH) in an NAD-dependent manner to generate phosphohydroxypyruvate which is transaminated and then dephosphorylated to generate serine. Serine hydroxymethyltransferase then donates the side-chain of serine to the Folate pool to generate glycine.

We quantified the relative amount of glycolytic flux being diverted into serine and glycine biosynthesis and found that it was comparable to the amount of glucose passing through glycolysis and ultimately being converted to lactate. However, this large flux was observed to be present in only a subset of cultured cells. A non-tumorigenic breast epithelial cell line was found to have no detectable flux into serine and glycine biosynthesis. The observation that some but not all cancer cells divert a large amount of glycolytic flux into serine and glycine metabolism suggested that there might be a context in which this flux might be selected for in the development of cancer.

To search for this context, we investigated a pooled analysis of 3131 human cancers [8]. This analysis revealed that PHGDH, the gene that encodes the first enzyme in this biosynthetic pathway branching off of glycolysis is frequently present in a region of recurrent, localized copy number gain at genomic locus 1p12 (q=1.18e-9). The amplification was most commonly found in melanoma but was observed in other cancers such as eosophageal adenocarcinoma and triplenegative breast cancer. We validated this finding in a collection of human melanoma tissue samples by comparing PHGDH expression in human tissues as measured by Immunohistochemistry with matched assessments of PHGDH copy number gain using fluorescence in situ hybridization. It was observed that PHGDH protein expression correlated with genomic copy number gain.

To assess whether cells containing the amplification were differentially sensitive to inhibition of PHGDH expression, we considered a panel of tumor-derived human Melanoma cell lines. Each melanoma cell line with PGHDH amplification exhibited significant flux into serine biosynthesis. We then carried out an RNA interference study and found that decreased PHGDH expression by RNA interference impaired growth in cell lines containing amplification of PHGDH and resulted in a distinct metabolic phenotype marked by accumulation of glycolytic intermediates.

PHGDH expression was also found to be associated with aggressive breast cancer subtypes. Since we observed no detectable flux in a non-tumorigenic breast epithelial cell line, we questioned whether enhanced PHGDH expression would have any phenotypic consequences.

We considered the effects of PHGDH in a model of breast tissue morphogenesis [9]. In this model, ectopic expression of PHGDH in MCF10A cells induced loss of cell polarity, disruptions in nuclear architecture and rescue of anoikis – each being phenotypic alterations that predispose cells to tumorgenicity. Our findings demonstrate that altered metabolic flux stemming from glycolysis can be selected for in the development of cancer and contribute to cell transformation. Furthermore, our studies identify PHGDH as an attractive therapeutic target for subsets of human cancers.
